# Investigation of Fenbendazole Solubility Using Particle Size Reduction Methods in the Presence of Soluplus^®^

**DOI:** 10.3390/pharmaceutics17091163

**Published:** 2025-09-04

**Authors:** Amirhossein Karimi, Pedro Barea, Óscar Benito-Román, Beatriz Blanco, María Teresa Sanz, Clement L. Higginbotham, John G. Lyons

**Affiliations:** 1 PRISM Research Institute, Technological University of the Shannon, N37 HD68 Athlone, Ireland; clem.higginbotham@tus.ie; 2 Department of Biotechnology and Food Science (Chemical Engineering Section), Faculty of Sciences, University of Burgos, Plaza Misael Banuelos s/n, 09001 Burgos, Spain; pbgomez@ubu.es (P.B.); obenito@ubu.es (Ó.B.-R.); bblanco@ubu.es (B.B.); tersanz@ubu.es (M.T.S.); 3 Faculty of Engineering & Informatics, Technological University of the Shannon, N37 HD68 Athlone, Ireland

**Keywords:** fenbendazole, Soluplus^®^, size reduction techniques, ultrasonication, microfluidization, solubility

## Abstract

**Background/Objectives**: Fenbendazole is a potential cancer treatment and a proven antiparasitic in veterinary applications. However, its poor water solubility limits its application. In this study, potential fenbendazole solubility enhancement was investigated through size reduction methods. The effect of the presence of Soluplus^®^ on solubility was investigated as well. **Methods**: Solubility enhancement was explored using microfluidization and ultrasonication techniques. These techniques were applied to fenbendazole alone and in combination with Soluplus^®^. UV–Vis spectroscopy was used to determine solubility. Possible chemical reactions were checked using Fourier transform infrared spectroscopy (FT-IR). Differential scanning calorimetry (DSC) was conducted to analyze the physical structure and crystallinity of the samples. Scanning electron microscopy (SEM) was also utilized for characterization of the effect of the treated formulations and the size reduction method on morphology. The elements present in samples were identified with energy-dispersive X-ray spectroscopy (EDX) combined with SEM. A comparison of crystalline structure between the products was performed via X-ray powder diffraction (XRPD). Dynamic light scattering (DLS) was also used to measure the samples’ average particle size at different stages. **Results**: Both ultrasonication and microfluidization led to marginal increases in the solubility of neat fenbendazole. In contrast, formulations processed in the presence of Soluplus^®^ demonstrated a greater enhancement in solubility. However, solubility improvement was not retained in the dried samples. The post-drying samples, irrespective of the presence of Soluplus^®^, showed nearly the same solubility as neat fenbendazole. **Conclusions**: Size-reduction methods, particularly when combined with Soluplus^®^, improved the solubility of fenbendazole. However, drying appeared to reverse these gains, regardless of the method used.

## 1. Introduction

Fenbendazole is an anthelmintic drug widely used in veterinary medicine [1,2]. In addition to its application as an antiparasitic medicine, it has a potential anticancer application, which was first offered in 2013 [3]. However, this application is limited by its poor water solubility (0.3 µg/mL), resulting in low bioavailability [4]. It is classified as a Class II drug under the Biopharmaceutics Classification System (BCS) [5]. Its solubility is pH-dependent and increases as the pH decreases [6].

Several strategies, including solid dispersions [7,8], complexation [9,10], salt formation [11], cosolvents [12,13], nanocrystallization [14], polymeric micelles [15], and hot melt extrusion [7], have been explored to overcome this limitation.

Although these methods have shown enhanced solubility, challenges such as poor long-term stability [16], multistep processing [14], and the presence of organic solvents [13] highlight the need for further investigation. In contrast, particle size reduction combined with polymer carriers such as Soluplus^®^ offers a practical and potentially scalable alternative for enhancing solubility while retaining formulation stability in solid form.

Soluplus^®^ is an amphiphilic graft copolymer (polyvinyl caprolactam–polyvinyl acetate–polyethylene glycol) that functions as a solubilizer and is widely used in pharmaceutical formulations to enhance the solubility and bioavailability of poorly water-soluble drugs. Its hydrophobic backbone interacts with poorly water-soluble drugs through hydrophobic and van der Waals interactions, while its hydrophilic side chains enhance wettability and aqueous dispersion [15].

The effect of Soluplus^®^ on the bioavailability of fenbendazole was investigated in an in vivo study, which showed that fenbendazole-loaded Soluplus^®^ had superior bioavailability to that of neat fenbendazole through micelle formation. The effect of Soluplus^®^ in wet milling (as a size reduction technique) on another poorly water-soluble drug (fenofibrate) was also investigated [17]. This study confirmed higher solubility in the presence of this solubilizer [17]. The addition of Soluplus^®^ to the system during processing may demonstrate its effect as an in situ solubilizer and could indicate a possible impact on solid dispersion stability after drying [15,18,19,20].

Particle size reduction has become one of the most prominent approaches to overcoming the solubility problems of poorly water-soluble drugs [21]. Since smaller particles provide a higher surface area of the drug, solubility increases [22,23,24].

Among various particle size reduction techniques, microfluidization has been developed as an important method because it produces uniform submicron- and nanoscale particles. This method uses high-pressure homogenization, where the drug suspension is subjected to very high shear forces that effectively reduce the particle size [25,26].

Ultrasonication is a size reduction technique that has shown promising results in some studies for certain insoluble active drugs. It involves the application of high-frequency sound waves to reduce particle size and enhance solubility [27,28,29,30].

Ultrasonication offers several key advantages regarding solid dispersion. It is considered an effective approach to enhancing pharmaceutical performance [31]. In addition, it can significantly enhance the solubility of poorly water-soluble drugs by improving wettability and dispersibility [32]. This treatment promotes homogeneity within drug–polymer systems at the molecular level, thereby facilitating stronger interactions and improved stability [33]. Moreover, ultrasonic waves reduce the particle size of drugs to nano or submicron ranges, thereby significantly increasing the surface area available for dissolution and breakdown [34,35].

Both ultrasonication and microfluidization are scalable technologies suitable for pharmaceutical production. Ultrasonication is relatively easy to scale up and, therefore, practical for industrial manufacturing processes in pharmaceutical formulation [36]. Microfluidization also enables continuous processing at high pressure, making both methods viable for industrial adoption [37]. However, ultrasonication may cause heat build-up and introduce metallic particles from the probe into the sample due to cavitation-induced abrasion. Meanwhile, high-pressure homogenization (microfluidization) may increase the risk of thermal or chemical degradation [38].

This study uses ultrasonication and microfluidization techniques to examine the effects of in situ methods in conjunction with Soluplus^®^ on fenbendazole, both prior to and following drying. A gap rarely investigated in fenbendazole studies is filled by presenting comparative data between these processing methods and drying strategies, such as freeze-drying and sample concentration. In particular, this study looks at how different ultrasonication durations and microfluidization cycles, as well as the application of Soluplus^®^ as a polymeric solubilizer, affect the solubility enhancement of fenbendazole. In contrast to earlier research that focused on improving solubility through solid dispersion, hot melt extrusion, or polymeric micelles, this study directly compares two scalable particle size reduction methods (microfluidization and ultrasonication) in the presence of Soluplus^®^. Crucially, this study also assesses the effects of different drying techniques (freeze-drying and sample concentration) on the maintenance or loss of solubility enhancement. At present, fenbendazole has not been the subject of a combined comparison specifically focusing on the drying process. The sample concentrator operates at a lower temperature than the boiling point, using a nitrogen evaporation system, which may affect morphology and crystallinity. In contrast, the freeze-dryer operates at much lower temperatures, potentially preserving the molecular structure and avoiding recrystallization [39]. These differences could influence solubility values, justifying the use of both methods for comparison [40]. The treated formulations were characterized using various analytical techniques, such as UV–Vis spectroscopy, to check the solubility after the dissolution test. DSC and FT-IR were employed to assess the physical and chemical characterization of the products. SEM, EDX, and XRPD were also conducted to check the crystalline structure and elemental composition of the samples.

## 2. Materials and Methods

### 2.1. Materials

Fenbendazole was purchased from Sigma-Aldrich, Darmstadt, Germany, and was used as received without further purification, with an assay of a minimum of 98%. Soluplus^®^ (polyvinyl caprolactam–polyvinyl acetate–polyethylene glycol graft copolymer) was purchased from BASF, Ludwigshafen, Germany. DMSO (dimethyl sulfoxide) 99.8+%, extra pure, was supplied by Thermo Fisher Scientific (Waltham, MA, USA) and used for preparation of the calibration curve. Deionized water was used for all experiments to eliminate potential contaminants or ions that could affect the results. The water was purified using a Milli-Q system (Merck Millipore, Burlington, MA, USA) to ensure high measurement accuracy.

### 2.2. Sample Preparation

Samples were prepared by adding 200 mg of fenbendazole to 200 mL of deionized water, stirring at 1000 rpm using a magnetic stirrer (model G71HS07C, supplied from OHAUS^®,^ Parsippany, NJ, USA) for 60 min at room temperature (RT). A total of 3 mL of the sample was filtered using 0.22 µm nylon syringe filters (obtained from Thermo Fisher Scientific (Waltham, MA, USA)) to assess the soluble content using UV–Vis. This process was conducted in triplicate to ensure repeatability.

Subsequently, 200 mg of fenbendazole was added to 200 mL of deionized water and mixed for 15 min with a magnetic stirrer at room temperature. After that, the mixture was further treated for 15 min via ultrasonication at 750 W, using 5 s on/5 s off cycles (30 min total), with a 20 kHz probe sonicator purchased from Fisher Bioblock Scientific (Illkirch, France). The temperature of the process was controlled using a double-layer chamber with a cooling pump at 25 ± 1 °C. To check the effect of ultrasonication time (consumed energy), three additional samples of the same concentration were prepared and subjected to ultrasonication for 30, 60, and 90 min (corresponding to a total treatment time of 60, 120, and 180 min). A total of 3 mL of each sample was collected at the end of the process and filtered. All experiments were performed in triplicate.

Samples sonicated for 60 min (corresponding to the highest solubility and optimum time of ultrasonication, as described in Section 3.3) were collected to study the effect of drying methods. A total of 50 mL of the sample was dried in 25 tubes of 2 mL under 40 °C and nitrogen, merged for 24 h, using a Cole-Parmer BH-250 sample concentrator obtained from Antylia Scientific, Chicago, IL, USA. Another 50 mL of the samples was also dried using a Telstar LyoQuest freeze-dryer (Terrassa, Spain) at −83 °C.

The same concentration of fenbendazole in water was used in the microfluidizer, model LM20, with an interaction chamber F20Y, from Microfluidics International Corporation, Westwood, MA, USA. The mixture was processed through the 2000-bar chamber for 5 cycles. Then, 3 mL samples were collected from each cycle and filtered with the same syringe filter to check the effect of the microfluidizer on the solubility of fenbendazole in water. A total of 50 mL of the final sample was dried by freeze-drying, and 50 mL was dried using the sample concentrator under the same conditions as before. This method was also performed three times to ensure reproducibility.

Dried samples were dissolved in a model 2100B DISTEK Inc., North Brunswick, NJ, United States, while the medium (water) was maintained under controlled conditions and temperature. Each sample was introduced to the batches at the same time to evaluate solubility. Samples of 3 mL were collected at regular intervals (0, 15, 30, 60, 120, 180, 240, 300, 360, and 1440 min) and replaced with fresh media. The collected samples were filtered using the same filters as before. Each sample was tested in triplicate to ensure reproducibility. Fenbendazole concentrations were measured using a calibration curve, which was subsequently employed to generate a graph illustrating the correlation between time and solubility.

All the procedures were repeated with the addition of 800 mg of Soluplus^®^ to 200 mg of fenbendazole. The composition of all samples is presented in Table 1. A 1:4 drug-to-polymer ratio was selected based on previous studies and literature reviews related to the fenbendazole in the solid dispersion method to investigate an acceptable ratio in the pharmaceutical industry and solid dispersion method [8,41,42].

### 2.3. Characterization Techniques

#### 2.3.1. DSC

The thermal properties of the samples were evaluated using a Pyris 6 DSC instrument (PerkinElmer, Inc., Shelton, CT, USA). Samples were placed in a vacuum oven at 80 °C for 18 h to remove thermal history and possible absorbed moisture. Approximately 6 mg of each sample was sealed in an aluminum pan for analysis. A constant nitrogen gas flow of 30 ± 1 mL/min was maintained throughout the analysis to prevent oxidation. An isothermal step of 1 min at 20 °C was applied to the samples to stabilize them and ensure a uniform temperature prior to the heating step. The heating rate of 10 °C/min from 20 °C to 300 °C was applied to all the samples. This method follows the literature review with some modifications [7,8]. The DSC data were analyzed using Pyris Manager Software (version 13.3.1.0014) to identify transition temperatures, and graphs were generated using Origin Pro 2022b software.

#### 2.3.2. Fourier-Transform Infrared (FT-IR) Spectroscopy

Spectra were recorded using a PerkinElmer Spectrum One spectrometer (PerkinElmer, Inc., Shelton, CT, USA). This instrument was equipped with a universal Attenuated Total Reflectance (ATR) accessory, enabling non-destructive analysis and simplified sample preparation. Spectra were collected over a wavenumber range of 400–4000 cm^−1^, with each sample analyzed in quadruplicate. A consistent compression force of 85 N was manually applied during the measurements on powdered and pelletized samples. Data processing and analysis were carried out using Spectrum software, IRPal v2.0, and Origin Pro 2022b, and the results were cross-validated with relevant literature.

#### 2.3.3. UV–Visible (UV–Vis) Spectroscopy

The solubility of fenbendazole was determined using UV–Vis spectroscopy (Hitachi U-2000, Tokyo, Japan), which measures the absorbance of fenbendazole solutions. Each sample was measured in triplicate using UV–Vis spectroscopy at 288 nm. Absorbance values were recorded up to three decimal places, and the mean values were used for solubility calculations according to the calibration equation.

The UV–Vis spectrophotometer was used to measure absorbance at 288 nm, as reported in the literature [8]. This device provided us with two cuvettes for each test, one for the reference solution containing water (or Soluplus^®^ solution of 8 mg/mL) and the other one as the samples containing fenbendazole. Additionally, to quantify the soluble drug, solutions of different concentrations (3, 4, 5, 6, 7, 14, 21, 28, 35, 42, 49, 56, 63, and 70 μg/mL) were prepared for a calibration curve with DMSO. Absorbance was recorded for samples collected after processing, and their solubility improvements were assessed by comparing them.

**Figure 1:**
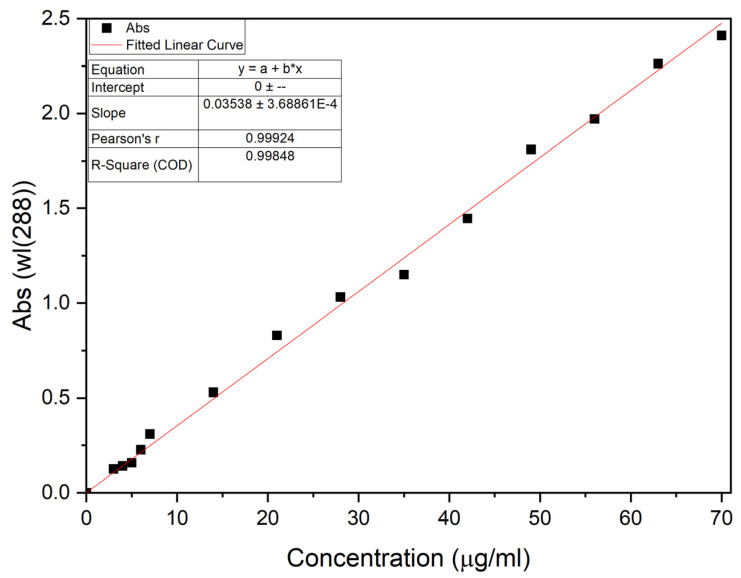
Calibration curve of fenbendazole solution in DMSO quantification using UV–Vis spectrophotometry.

The calibration curve was constructed using the standard fenbendazole solution in DMSO (Figure 1). The linear character of the calibration curve supported the accuracy of absorbance measurement in dissolution tests [43]. The characteristic of the fitted linear curve was calculated using Origin Pro 2022b and is presented in Figure 1.

Based on information of the fitted curve, we can use Equation (1) to identify the fenbendazole concentration in each sample.

(1) $$Fenbendazole\;Concentration=\frac{Average\;absorbance\;at\;288\;\mathrm{nm}}{0.03538}$$

#### 2.3.4. SEM, EDX

The morphology and particle size of fenbendazole before and after preparation were examined using SEM. Images were acquired using a SEM (TESCAN MIRA2, Brno, Czech Republic) at an accelerating voltage of 5–20 kV. The samples were mounted on aluminum stubs and coated with gold (Au) with a 10 nm thickness to render them electrically conductive. The effects of different drying techniques (freeze-drying and sample concentration) and processing methods (ultrasonication and microfluidization) were analyzed in terms of particle size reduction and surface structure. The elemental composition was characterized by EDX with the same SEM device and samples. The EDX spectra were recorded at the same accelerating voltage, and all the detected elements were analyzed. However, sulfur as the primary element was monitored for recognition of fenbendazole due to its characteristic role in the chemical structure.

#### 2.3.5. XRPD

Powdered samples were analyzed using a PANalytical Aeris Instrument Suite (Malvern Panalytical Ltd., GmbH, Kassel, Germany). The instrument was equipped with Cu Kα radiation (λ = 1.54078 Å), operating at 30 kV and 10 mA. The experiments were conducted using a 1/8° divergence slit, a Ni β-filter, a 0.04° Soller module, and a Pixcel^1D^-Medipix3 detector. The samples were mounted on a silicon single-crystal zero-background sample holder and examined in reflection mode while spinning at four rotations per second. Data were collected over a 2θ range of 5–40° with a step size of 0.021°.

#### 2.3.6. DLS

The particle size distribution of the samples (containing Soluplus^®^, fenbendazole, and their combinations) before size reduction, after processing, and following drying was measured using the DLS method with a Zetasizer Pro, Malvern Panalytical, Worcester, Worcestershire, UK. All the measurements were performed in DTS0012 disposable polystyrene cuvettes and conducted at 25 °C, with the instrument’s temperature equilibration time set to 60 s to ensure thermal stability. The water dispersant’s physical properties (refractive index = 1.33; viscosity ≈ 0.8872 mPa·s at 25 °C) were input into the ZS Xplorer v1.0.0 software. The fenbendazole refractive index was set as 1.674 according to the literature [44].

## 3. Results

### 3.1. DSC

**Figure 2:**
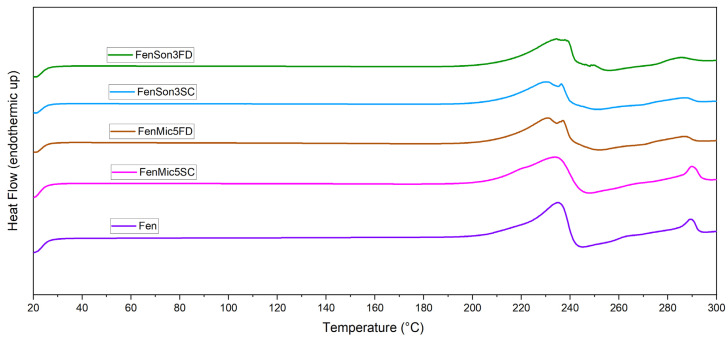
Thermal behavior of fenbendazole and modified fenbendazole samples through ultrasonication or microfluidization, freeze-dried or dried by the sample concentrator, through DSC from 20 to 300 °C.

**Figure 3:**
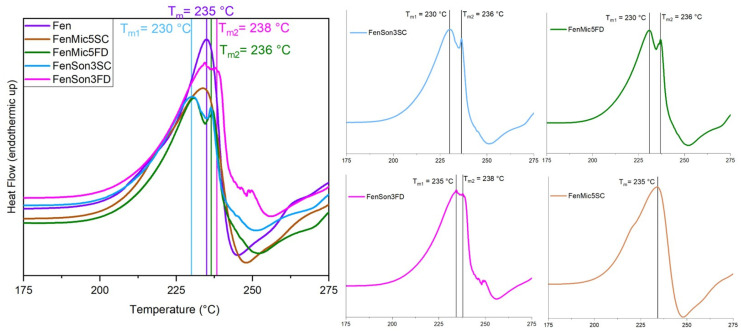
Focused view of thermal behavior of fenbendazole and modified fenbendazole samples in the melting temperature range through DSC (175 to 275 °C). The left panel shows the overlaid DSC curves for pure fenbendazole and modified fenbendazole samples prepared by microfluidization (FenMic5SC and FenMic5FD) and ultrasonication (FenSon3SC and FenSon3FD). The right panels present the individual DSC curves for each modified sample. The temperature is indicated by vertical lines, highlighting differences between the T_m1_ and T_m2_ as observed in certain samples.

**Figure 4:**
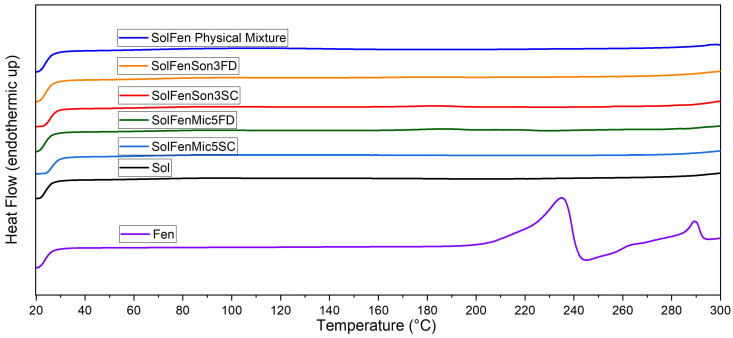
Thermal behavior of fenbendazole, Soluplus^®^, and modified fenbendazole samples included Soluplus^®^ through ultrasonication or microfluidization and freeze-dried or dried by the sample concentrator through DSC from 20 to 300 °C.

**Figure 5:**
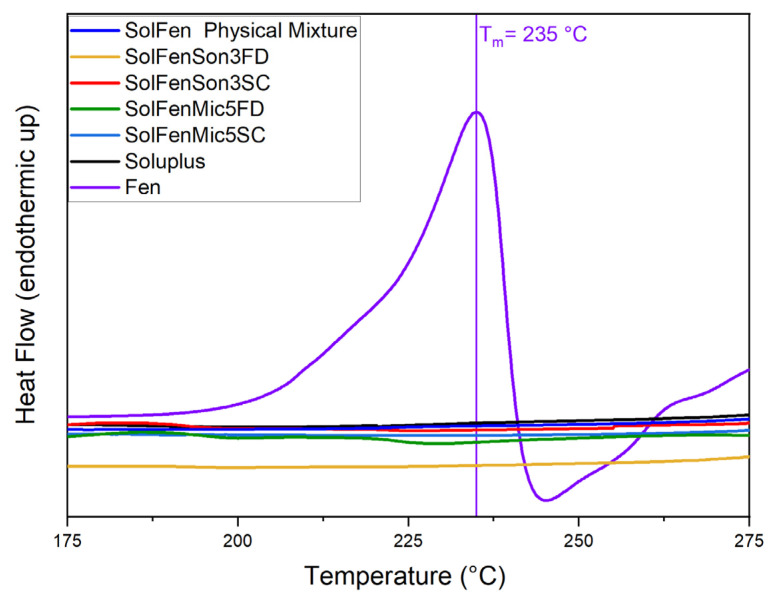
Focused view of thermal behavior of fenbendazole, Soluplus ^®^, their physical mixture, and modified fenbendazole–Soluplus^®^ samples in the melting temperature range through DSC (175 to 275 °C).

**Figure 6:**
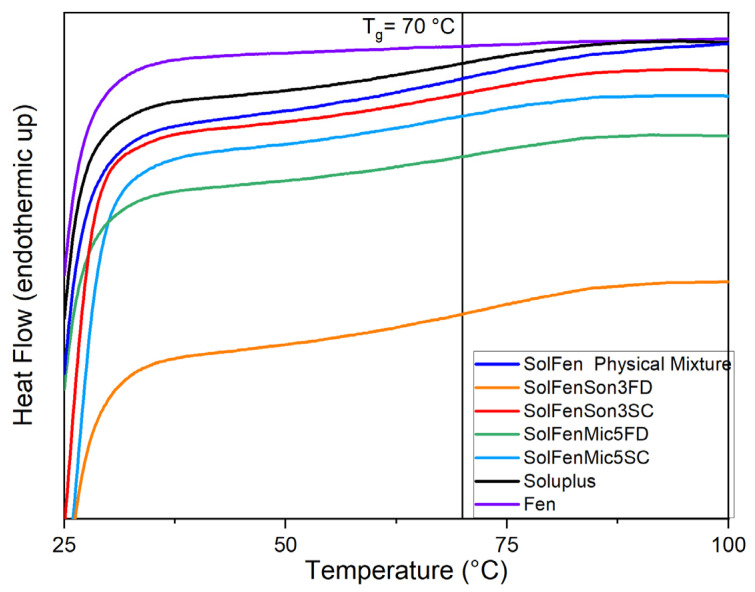
Focused view of thermal behavior of fenbendazole, Soluplus ^®^, their physical mixture, and modified fenbendazole–Soluplus^®^ samples in the glass transition temperature range through DSC (25 to 100 °C).

The thermal behavior of the samples was evaluated through DSC, and the obtained thermograms provide details on phase transitions, crystallinity, and thermal stability. The DSC curves for the studied samples are presented in Figure 2, Figure 3, Figure 4, Figure 5 and Figure 6, highlighting thermal phenomena such as the glass transition temperature (T_g_), the melting temperature (T_m_), and crystallization features. Figure 2 illustrates that the modification processes on samples that include just fenbendazole did not cause any changes in the overall trend of the curves. This indicates that the thermal behavior of the samples did not exhibit any noticeable change. Additionally, Figure 3 focuses on the T_m_ area and accurately shows that the T_m_ of processed samples is within a close range of the T_m_ value of fenbendazole (235 °C) for all the samples, which is from 230 °C to 238 °C. Rather than a change in crystal structure, the small variation in peak melting temperature (within ~5 °C) is attributed to differences in crystallite size or defect density. This is consistent with the literature, where the melting peak remained constant when the crystalline structure was preserved [45,46]. In contrast, Figure 3 shows that the peak area of the modified fenbendazole samples decreased marginally. This area indicates the enthalpy of fusion, which represents the required energy to overcome the intermolecular forces that maintain the crystalline structure [47].

The degree of crystallinity (X_c_) was ascertained by the enthalpy of fusion (ΔH_f_), through Equation (2), where ΔH_f_ is the measured enthalpy of fusion, and ΔH_f_˚ represents the enthalpy of fusion for fully crystalline fenbendazole [48].

X_c_ ₌ ΔH_f_/ΔH_f_˚ (2)

Figure 3 also indicates that three of the samples show two peaks in the T_m_ range, which may indicate some accrued defects in the structure of certain crystallites. These points are named T_m1_ and T_m2_. The collected DSC results, related to the fenbendazole and processed fenbendazole samples, including T_m1_, T_m2_, ΔH_f_, and X_c_, are presented in Table 2.

The results indicated that the crystallinity of the processed sample decreased compared to neat fenbendazole. This reveals that both ultrasonication and microfluidization can decrease the required energy for melting and crystallinity, probably due to the particle size reduction capability of these techniques [49]. The reduction in crystallinity was more pronounced in samples processed through microfluidization compared to those subjected to ultrasonication. X_c_ and ΔH_f_˚ also showed noticeably lower values when samples were dried through the sample concentrator.

Figure 4 shows the overall thermal behavior of fenbendazole, Soluplus^®^, their physical mixture, and the modified samples in the presence of Soluplus^®^. The general trends of curves related to the modified samples and physical mixture follow the same trend as Soluplus^®^. Figure 4 does not show any first or second-order transition, which, respectively, are related to T_m_ and T_g_ in the samples. The T_g_ of Soluplus^®^ was reported at approximately 60–80 °C in the literature, while the melting peak was never observed, which indicates the amorphous nature of the Soluplus^®^ polymeric matrix [50]. Figure 5 and Figure 6 focus in depth on the melting and T_g_ range.

Figure 5, focusing on the T_m_ range, shows that in the modified samples in the presence of Soluplus^®^, regardless of the size reduction method, the melting peak disappeared. This phenomenon occurs in the fenbendazole–Soluplus ^®^ physical mixture as well. The reason for this behavior may be the dilution of fenbendazole in Soluplus^®^, which can happen during the heating process in the DSC test. This phenomenon has been noted to influence the crystal structure of fenbendazole [8,51].

Figure 6, focusing on the glass transition range, illustrates that the T_g_ was not shifted in modified samples, including the active drug portion and Soluplus^®^, which indicates that fenbendazole does not affect the polymer chain structure of Soluplus^®^ [52]. It is also evident that the second-order transition (T_g_) is not observed in the fenbendazole sample.

DSC analysis indicates that ultrasonication and microfluidization slightly decrease crystallinity, while the incorporation of Soluplus^®^ significantly influences thermal behavior, especially crystallinity and enthalpy of melting in the compounds, suggesting its suitability to increase solubility [8].

### 3.2. FT-IR Spectroscopy

**Figure 7:**
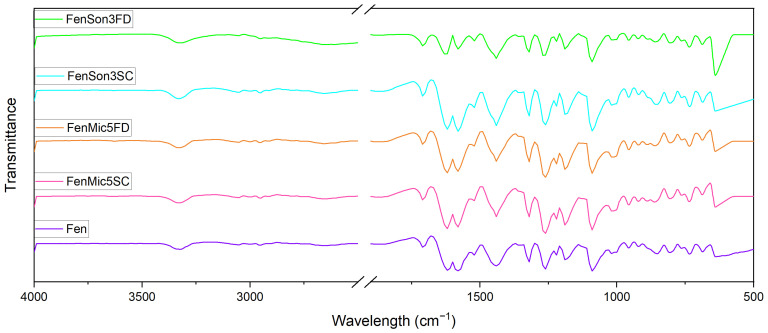
FT-IR spectra of fenbendazole and modified fenbendazole samples processed via ultrasonication or microfluidization and subsequently either freeze-dried or dried using the sample concentrator. The wavelength range from 2500 to 1900 cm^−1^ has been omitted, as it contains no relevant information.

FT-IR was conducted for chemical structure analyses of the individual components, fenbendazole and Soluplus^®^, and the products. The aim was to assess any possible chemical interaction due to their mixture. The FT-IR spectra obtained for fenbendazole and modified fenbendazole samples are presented in Figure 7. This figure shows that the process does not induce any noticeable changes in the chemical bonds and structure consistent with literature reports [53,54].

The FT-IR spectrum of fenbendazole (Figure 7) showed peaks characteristic of the fenbendazole molecular structure. This included the more prominent absorption peaks representing N-H stretching near 3340 cm^−1^ pertaining to amide function [55], C=O stretching vibration around 1620 cm^−1^ [56], and C-N stretching of benzimidazole around 1260 cm^−1^, in addition to peaks associated with aromatic C-H stretching around 3050 cm^−1^ with low absorbance, corresponding to benzene rings in the molecular structure of fenbendazole [57].

**Figure 8:**
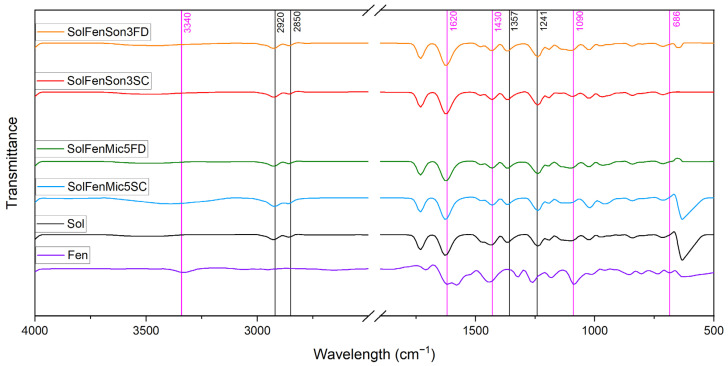
FT-IR spectra of fenbendazole, Soluplus^®^, and modified fenbendazole–Soluplus^®^ samples. Black lines indicate characteristic peaks of Soluplus^®^, and pink lines indicate characteristic peaks of fenbendazole. The wavelength range from 2500 to 1900 cm^−1^ has been omitted from the graph, as it contains no relevant information.

Figure 8 shows the FT-IR spectrum of fenbendazole, Soluplus^®^, and their mixed modified samples. The Soluplus^®^ spectrum in Figure 8 has characteristic peaks, including a broad O-H stretching vibration near its polymeric structure; C=O stretching at 1730 cm^−1^ [58], corresponding to ester groups in the structure of Soluplus^®^; and peaks between 1100 and 1300 cm^−1^ attributed to C-O-C stretching, confirming ether linkages present in the polymer backbone [53].

The absence of new peaks or significant shifts in the spectra aligns with earlier studies, indicating that fenbendazole incorporates into Soluplus^®^ without any chemical alteration. This is very advantageous in the context of drug delivery, since it implies that Soluplus^®^ can act as a carrier matrix and not interfere with the drug’s molecular integrity [59].

Figure 8 suggests the coating effect of Soluplus^®^ over fenbendazole particles. However, N-H stretching bonds in fenbendazole, which appear at 3340 cm^−1^, are observed over a broader range from 3200 to 3700 cm^−1^ in the fenbendazole–Soluplus^®^ samples. This peak shift may suggest that hydrogen bonding is a nonchemical effect of the Soluplus^®^ matrix on fenbendazole [60].

### 3.3. UV–Vis Spectroscopy

**Figure 9:**
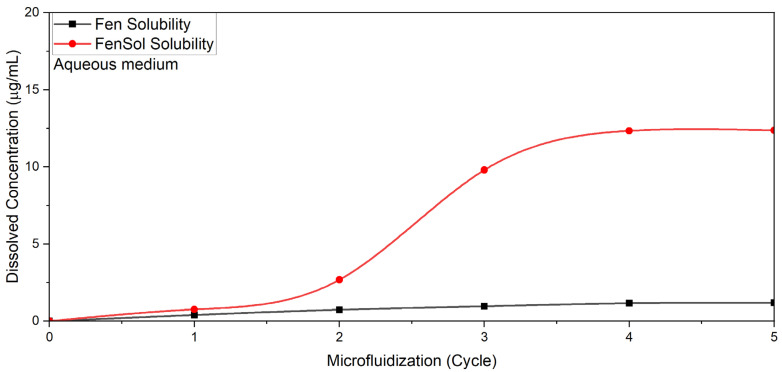
Effect of microfluidization on the solubility of fenbendazole and fenbendazole–Soluplus^®^ (1:4) formulations in water during five cycles of microfluidization.

Microfluidization exerted distinct effects on the dissolution characteristics of both fenbendazole and fenbendazole–Soluplus^®^ (20–80) systems. The results were positive in both cases. As presented in Figure 9, the modified samples exhibited a small increase in solubility due to particle size reduction and increased surface area, allowing for better interaction with the dissolution medium (water). However, the improvement was minimal (1.187 μg/mL), approximately four times higher than neat fenbendazole (which is 0.3 μg/mL), suggesting that particle size reduction alone is insufficient to enhance solubility appreciably [61]. The modified formulations containing Soluplus^®^ showed an increased dissolution to ~12.4 μg/mL (which is around 40 times more than neat fenbendazole), likely due to the stabilizing effect of Soluplus^®^ in dispersion and anti-agglomeration. These findings suggest the ability of Soluplus^®^ to maintain nano- and micro-sized particles before drying [62,63].

Table 3 shows the average UV absorbance and corresponding solubility values of fenbendazole and fenbendazole–Soluplus^®^ samples in water during the microfluidization cycles. A small difference between the solubility values between samples processed for four and five cycles through the microfluidization method suggests that five cycles may represent the optimal condition for this method.

**Figure 10:**
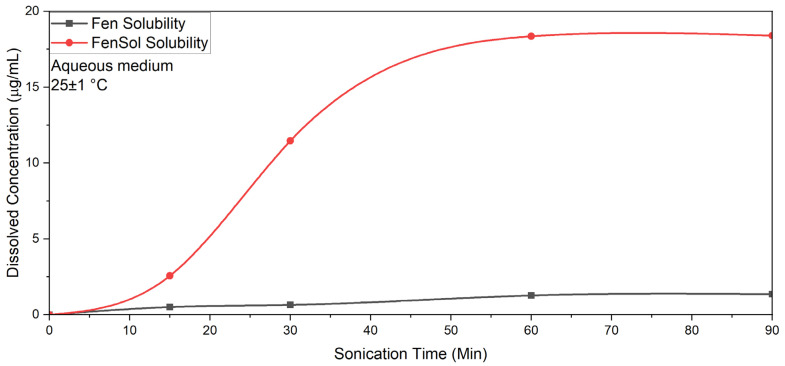
Effect of ultrasonication on the solubility of fenbendazole and fenbendazole–Soluplus^®^ (1:4) formulations in water during ultrasonication at 25 ± 1 °C.

Similar to microfluidization, ultrasonication enhanced dissolution by breaking apart aggregates and reducing the particle size. According to Figure 10, the solubility of fenbendazole under ultrasonication slightly increases to higher values (1.356 μg/mL). The solubility of formulated fenbendazole–Soluplus^®^ samples was 48% higher than that processed via microfluidization (18.4 μg/mL), representing more than 60-fold improvement in solubility compared to neat fenbendazole.

A comparison of solubility values between ultrasonication (Table 4) and microfluidization (Table 3) shows that ultrasonication can improve the solubility of all samples more effectively than microfluidization, specifically the samples that contain Soluplus^®^ as solubilizer. The solubility values also show a reverse trend compared to DSC results, where microfluidized samples exhibited a lower degree of crystallinity than ultrasonicated samples (Table 2).

Multiple factors associated with size reduction methods can increase the solubility of fenbendazole, such as increased surface area and decreased crystallinity. Since the solubility in the ultrasonicated samples is partially higher than microfluidized samples, despite a higher degree of crystallinity, increased surface area is a possible reason.

**Figure 11:**
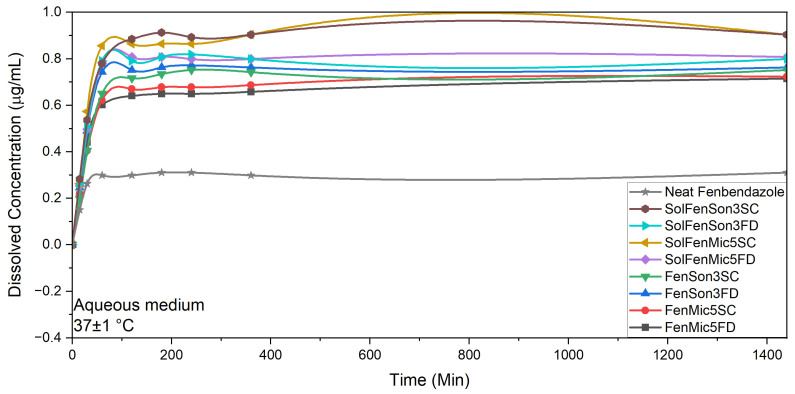
Effect of ultrasonication, microfluidization, drying method, and presence of Soluplus^®^ on the solubility of post-drying fenbendazole and modified samples during 24 h (1440 min) in water at 37 ± 1 °C. When the three replicate readings were identical, SD = 0. For other samples showing small variation among replicates, the SD was 1.6 × 10^−2^, consistent across all sets except those with SD = 0. Error bars were omitted for clarity.

One of the observations was the post-drying solubility reduction of all samples, regardless of the enhanced initial solubility following microfluidization and ultrasonication, or different drying methods (Figure 11). The solubility study of dried and subsequently re-analyzed processed samples showed marginally enhanced solubility compared to neat fenbendazole regardless of Soluplus^®^ presence. The average solubility values and average UV absorbance at 288 nm, of post-drying samples and neat fenbendazole are presented in Table 5 and Table 6, respectively, for better comparison.

Sample standard deviations (SDs) were calculated using Origin Pro 2022b. When the three replicate readings were identical, SD = 0. For other samples showing small variation among replicates, the SDs were 5.8 × 10^−4^ for UV absorbance and 1.6 × 10^−2^ for solubility. In other words, SD values were the same for all the sets of samples, except for samples with SD = 0. Consequently, error bars were omitted from Figure 11 and Table 5 and Table 6 to maintain clarity and readability.

This observation may suggest that fenbendazole is reaggregated after drying. It may also suggest losing some of the amorphous content responsible for initially enhancing dissolution through recrystallization. It also confirms the possible better compatibility between fenbendazole and Soluplus^®^ at a higher drying temperature (40 °C in the sample concentrator compared to freeze-drier), according to the higher solubility values in samples containing Soluplus^®^ and dried using the sample concentrator. These results suggest that both processing methods may enhance dispersion but not necessarily prevent fenbendazole recrystallization, strengthen polymer interaction, and promote fenbendazole particle reaggregation.

### 3.4. SEM

**Figure 12:**
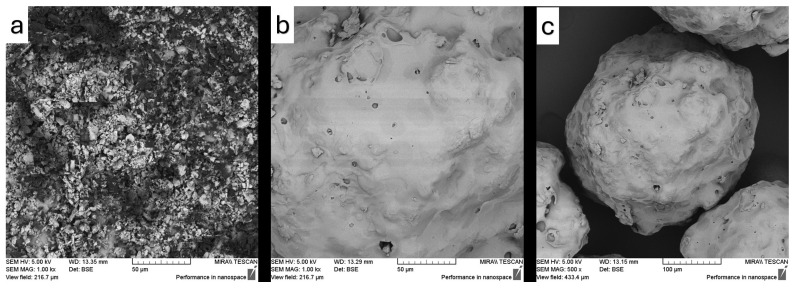
SEM images of (**a**) neat fenbendazole particles (Fen), 1000× magnification; (**b**) neat Soluplus^®^ single particle (Sol), 1000× magnification; and (**c**) neat Soluplus^®^ single particle (Sol), 500× magnification.

**Figure 13:**
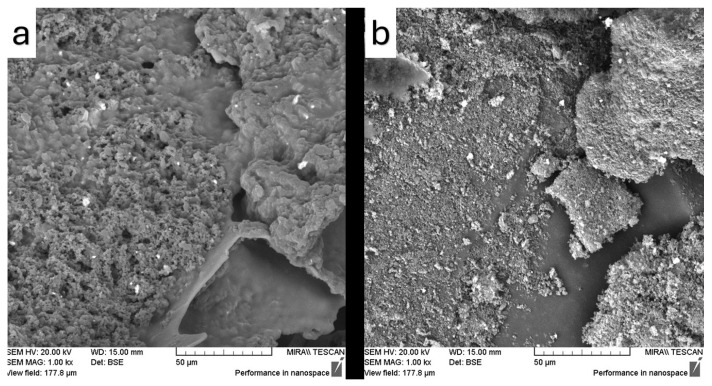
SEM images of (**a**) ultrasonicated fenbendazole dried using the sample concentrator (FenSon3SC), 1000× magnification; and (**b**) ultrasonicated fenbendazole, which was freeze-dried (FenSon3FD), 1000× magnification.

**Figure 14:**
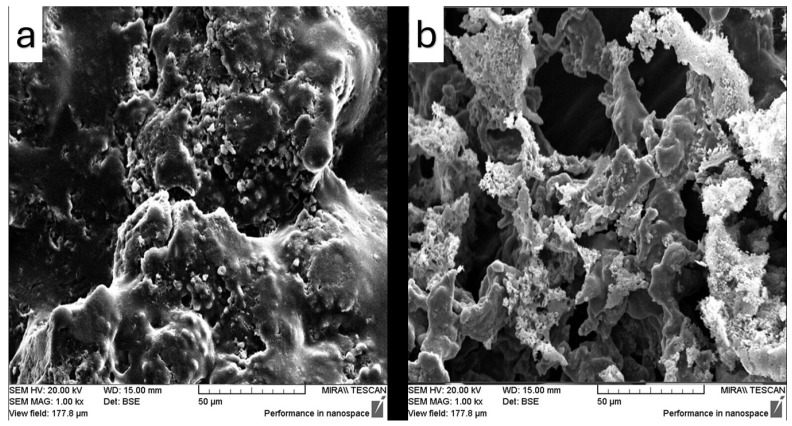
SEM images of (**a**) microfluidized fenbendazole dried using the sample concentrator (FenMic5SC), 1000× magnification; and (**b**) microfluidized fenbendazole, which was freeze-dried (FenMic5FD), 1000× magnification.

**Figure 15:**
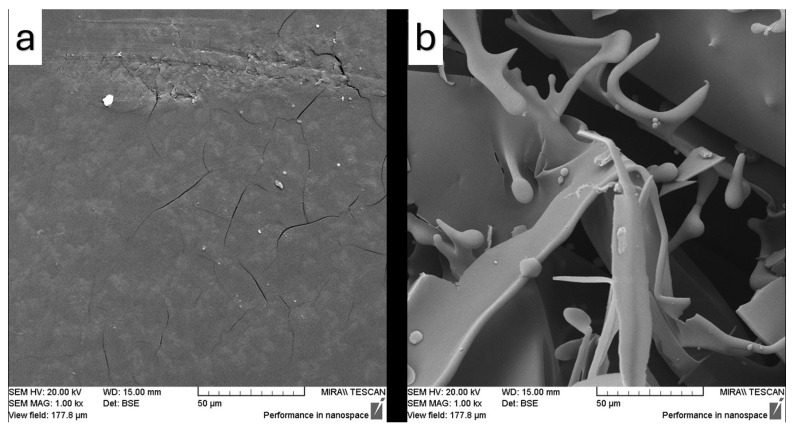
SEM images of (**a**) ultrasonicated formulation dried using the sample concentrator (SolFenSon3SC), 1000× magnification; and (**b**) ultrasonicated formulation, which was freeze-dried (SolFenSon3FD), 1000× magnification.

**Figure 16:**
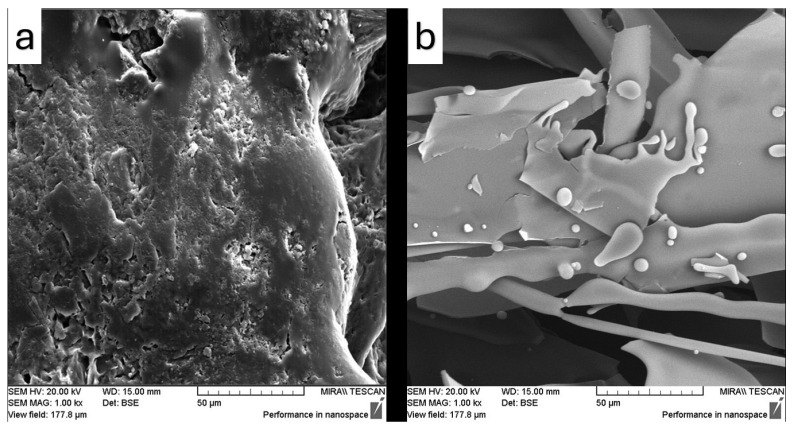
SEM images of (**a**) microfluidized formulation dried using the sample concentrator (SolFenMic5SC), 1000× magnification; and (**b**) microfluidized formulation, which was freeze-dried (SolFenMic5FD), 1000× magnification.

The surface microstructure and microscopic features of the samples were examined using SEM, and the resulting images are presented in Figure 12, Figure 13, Figure 14, Figure 15 and Figure 16. SEM micrographs show pronounced differences in the surface texture, particle orientation, and structure in the samples under examination.

Figure 12a contains the SEM image of Fen, which shows a rough surface with sharp edges, indicative of its crystalline structure of neat fenbendazole. However, the Soluplus^®^ (Figure 12b) demonstrates a relatively smooth, uniform, and curvy surface. Figure 12c also shows Soluplus^®^ at a lower magnification, revealing the spherical geometry and smooth morphology of its particles.

Figure 13 illustrates the effect of the ultrasonication and drying method on the fenbendazole structure and geometry after drying. Figure 13a shows that the surface appears smoother with increased porosity in the structure, which can be the result of the ultrasonication cavitation and drying method (sample concentrator). Furthermore, Figure 13b shows a smooth surface with smaller discrete particles, which can be the result of freezing after ultrasonication. Figure 14 demonstrates comparable trends through the microfluidizer device, while ultrasonication produced a smoother surface compared to the microfluidization method. Figure 14b also shows larger structures, most likely due to the freeze–drying method, while Figure 14a suggests more surface area based on the observed size reduction.

Figure 15 presents the SEM images of the samples containing Soluplus^®^ during the ultrasonication modification. Figure 15a also shows a brittle morphology characterized by surface cracks, which is an expected behavior of Soluplus^®^. However, this image illustrates a very smooth surface after using the sample concentrator. It also shows some small particles observed on the surface, which may indicate fenbendazole reaggregation, but the particles appear to be covered by Soluplus^®^. On the other hand, Figure 15b shows a higher surface area due to the porosity, which can be achieved through the freeze-drying method in the presence of a polymeric matrix. Larger aggregated particles are also observed when using this method; however, they seem to be covered by Soluplus^®^. Figure 16 similarly indicates that both microfluidization and ultrasonication can exert a comparable influence on surface microstructure, geometry, and solubility.

### 3.5. EDX

**Figure 17:**
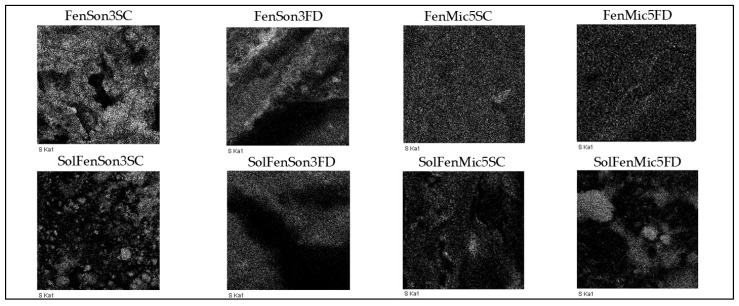
EDX images show sulfur in modified samples containing fenbendazole.

EDX coupled with SEM was employed to determine the elemental composition of the samples. Table 7 presents the EDX results, which summarize the elemental weight percentages for each sample. The EDX images of the samples confirmed the presence of characteristic elements, including carbon (C) and oxygen (O). Additionally, the incorporation of fenbendazole introduced sulfur (S) consistent with its chemical structure. Fenbendazole contains sulfur, so its presence is expected in the samples containing fenbendazole. Sulfur tracking by EDX images in the samples containing fenbendazole is shown in Figure 17.

Comparative elemental distribution analysis revealed that the active drug was heterogeneously distributed within the sample. There were higher concentrations of sulfur in some areas, suggesting a non-uniform dispersion. The presence of other elements, such as sodium (Na), potassium (K), calcium (Ca), chlorine (Cl), and titanium (Ti), likely indicates minor contamination during ultrasonication or microfluidization [64]. Additionally, gold (Au) was detected in all samples due to the sample coating. Table 7 illustrates the following elements and their weight percentages: carbon (C), oxygen (O), and sulfur (S), along with other detected elements mentioned as “others”.

### 3.6. XRPD

**Figure 18:**
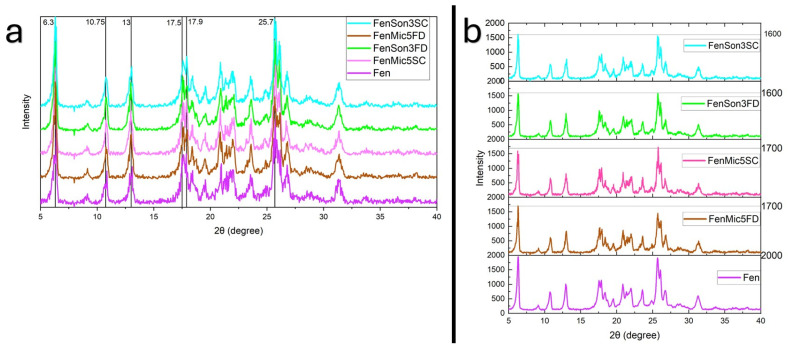
XRPD results of fenbendazole and its post-drying processed samples. (**a**) Comparison of X-ray diffraction of fenbendazole and modified samples through different size reduction and drying methods; (**b**) comparison of the intensity of X-ray diffraction of fenbendazole and modified samples through different size reduction and drying methods.

**Figure 19:**
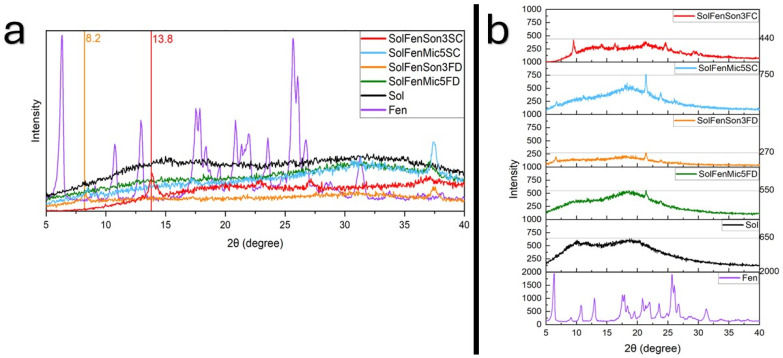
XRPD results of fenbendazole, Soluplus^®^, and their post-drying processed samples; (**a**) comparison of the X-ray diffraction degree of fenbendazole, Soluplus^®^, and modified samples through different size reduction and drying methods. The red line at 13.8° is related to the small peak in the plot that belonged to SolFenSon3SC, and the orange line at 8.2° is related to the small peak in the plot that belonged to SolFenSon3FD. (**b**) Comparison of the intensity of fenbendazole, Soluplus^®^, and modified samples through different size reduction and drying methods.

The composition of crystalline phases and the structure of samples were analyzed using XRPD. Test sample diffractograms are shown in Figure 18 and Figure 19, reflecting peaks in comparison with each other. Diffraction patterns provide hints of the changes in the degree of crystallinity, structural changes, and interactions among the ingredients.

Fenbendazole XRPD diffractogram in Figure 18 and Figure 19 exhibited characteristic diffraction peaks at different 2θ values, confirming its crystalline nature and different structures. Sharp and high-intensity peaks are indicative of a well-ordered crystalline nature, whereas broad or diffused peaks indicate an amorphous nature of Soluplus^®^ (Figure 16) [65]. Figure 18a indicates a lack of peak shifting in the results, which suggests that the crystalline structure of fenbendazole remains unchanged despite size reduction [65,66,67]. Figure 18b shows that size reduction methods, regardless of different drying methods, can lead to some partial changes in the intensity, which can be because of size reduction, since smaller particles may decrease the diffraction of X-rays [67]. A marginal peak broadening is also observed in Figure 18a, which confirms the size reduction possibility [65].

Upon the incorporation of Soluplus^®^ (Figure 19), distinct changes were observed in diffraction patterns. In drug–polymer systems, the absence of characteristic peaks of fenbendazole in the XRPD pattern of the composite sample suggests efficient molecular dispersion in the polymer matrix and supports the formation of an amorphous solid dispersion. The appearance of residual crystalline peaks in ultrasonicated samples (Figure 19a) suggests incomplete amorphization or phase separation [66].

The comparison between observed peak intensity in the samples produced through ultrasonication and microfluidization (Figure 19b) suggests that ultrasonication can be more effective in increasing the solubility of fenbendazole. It may also suggest the coating effect of Soluplus^®^ due to its bigger particles. The degree of crystallinity based on XRPD data can be calculated using Equation (3).

X_c_ = A_P_/A_T_ (3)

where X_c_ is the degree of crystallinity, A_P_ is the area under the crystalline peaks, and A_T_ is the area under the total diffractogram [68]. Data were calculated using Origin Pro 2022b. The resulting values are summarized in Table 8

Overall, XRPD analysis confirms that the structural properties were significantly altered by modifications in the presence of Soluplus^®^, whereas they were altered only partially for fenbendazole itself. The possible hydrogen bonds between Soluplus^®^ and fenbendazole may be the reason for the disappearance of the peaks, as indicated in the literature.

### 3.7. DLS

**Figure 20:**
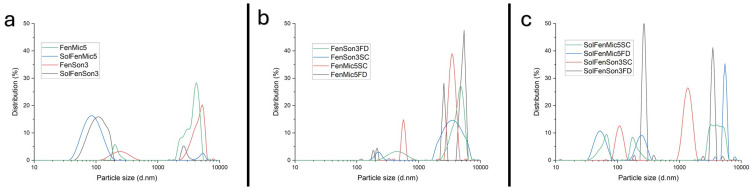
Particle size distribution of (**a**) fenbendazole and Soluplus^®^ formulation after size reduction techniques, (**b**) size-reduced fenbendazole samples after drying using the sample concentrator and freeze-dryer, (**c**) size-reduced fenbendazole–Soluplus^®^ samples after drying using the sample concentrator and freeze-dryer.

Results obtained from the DLS measurements demonstrate that the average particle size of the formulations ranged from approximately 300 nm to over 5000 nm, depending on the sample composition and processing conditions, according to Table 8 and Figure 20a–c. The PDI values varied considerably across the samples. Several formulations exhibited narrow size distributions (PDI < 0.3), while others showed much higher PDI values. All the data were collected from the Zetasizer, and the mean particle size and PDI were calculated using Origin Pro 2022b.

The results indicate that ultrasonication is more efficient than microfluidization in reducing the particle size. This finding may suggest that fenbendazole solubility is more dependent on particle size and increased surface area. Data presented in Table 9 also indicates that the presence of Soluplus^®^ can improve solubility during the size reduction process and increase stability in post-drying samples. Certain samples (e.g., SolFenMic5, SolFenSon3, SolFenMic5FD, and SolFenSon3FD) had PDI values higher than 1. The bimodal or trimodal and broad distribution seen in the particle size measurements was indicated by high PDI values. Variations in particle aggregation during microfluidization or ultrasonication and drying methods may be the cause of such wide distributions [69].

## 4. Discussion

The thermal behavior of the pure and formulated samples was investigated through DSC analysis. The results suggest that Soluplus^®^ can improve the thermal stability of modified samples [70]. This could result from increasing the activation energy of degradation according to the compatibility of Soluplus^®^ and fenbendazole [8,67,71].

In another study, the DSC thermograms of fenbendazole with Pluronic^®^ F-127 (P-407), poly-caprolactone (PCL), and poly (lactic acid) (PLA) revealed a reduction in the melting peak intensity and enthalpy of fusion, indicating a decrease in the crystallinity of solid dispersed fenbendazole samples. These changes indicate partial solubilization of fenbendazole in the polymer matrices and reduced crystallinity [8]. However, our research showed nearly complete solubilization of fenbendazole in Soluplus^®^ through DSC analysis, which results in the disappearance of the melting point peak in the Figures.

Although these results may suggest potential physical or chemical interaction between the components and suitability of the process to increase solubility [8], the FT-IR spectrum showed no chemical reaction. However, the possible formation of hydrogen bonds was indicated by the changes observed in the N-H stretching. The other study showed possible hydrogen bonds between fenbendazole and the PEO/PCL matrix, as evidenced by the disappearance or reduced intensity of (COO) and (C-O) peaks in the PCL FT-IR spectrum [7].

The results indicate that the mixture is a physical blend where both fenbendazole and Soluplus^®^ maintain their unique molecular structures without any chemical modification or degradation [8,72].

The FT-IR and DSC results established the potential of Soluplus^®^ as a polymeric carrier for fenbendazole in solid dispersion formulations or other drug delivery systems to preserve the drug’s integrity and stability within the formulation. However, the dissolution test results revealed only minimal enhancement of solubility in post-drying samples.

These observations suggest possible recrystallization during drying [73], inadequate polymer–drug interactions [73] or possible reaggregation of particles, reducing the effect of particle size reduction [74].

SEM images showed similar effects for both microfluidization and ultrasonication techniques on morphology, as evidenced by comparable surface microstructures of the products. The combination of SEM and EDX analyses provided valuable insights into the microstructural changes induced by size reduction and drying methods in the final product microstructure, confirming suboptimal dispersion, limited compatibility, and incomplete particle size reduction. Such findings confirm the suitability of the processing method for size reduction and are consistent with previous reports in the literature.

XRPD patterns revealed lower peak intensity in processed fenbendazole samples than in neat fenbendazole, indicating reduced crystallinity. However, the results showed that ultrasonicated samples appear to have lower crystallinity, a finding not fully corroborated by DSC analysis. This can be attributed to the fundamental principles and limitations of each technique. Particle size effects may reduce the observed enthalpy of melting, as smaller crystallites exhibit lower ΔH_f_ due to surface energy contributions and imperfect lattice ordering [68]. On the other hand, peak broadening and intensity reduction in fenbendazole-Soluplus^®^ diffraction patterns indicate crystallinity loss due to the dispersion of molecules or disruption of the ordered system [65,66]. This can be due to intermolecular interactions between fenbendazole and Soluplus^®^ matrix, leading to partial amorphization, as also supported by DSC findings. Another study on fenbendazole solid dispersion mixed with PEO/PCL diffraction pattern showed that solid dispersion could eliminate the crystalline structure; however, the maximum solubility achieved was only 5% after 24 h in a phosphate-buffered solution [7].

DLS test results showed that the post-drying samples’ particle size increased due to reaggregation of fenbendazole particles, while the mean particle size of the samples before drying was significantly smaller. The presence of Soluplus^®^ resulted in much smaller particles, but the particle size of post-drying samples indicates that Soluplus^®^ could not prevent fenbendazole reaggregation. In other words, the presence of Soluplus^®^ improves the stability of fenbendazole particles in aqueous medium after size reduction but cannot fully prevent reaggregation during drying.

These findings suggest that more optimized ratios, formulations, and strategies are required to guarantee long-term solubility enhancement of fenbendazole. It is also suggested that further in vivo studies should focus specifically on pre-drying samples under various physiological conditions. Additionally, exploring the methods and formulations to prevent reaggregation represents a crucial future direction.

## 5. Conclusions

This study investigated how microfluidization and ultrasonication affect the solubility, thermal, physical and chemical properties, and structural characteristics of fenbendazole itself and when combined with Soluplus^®^. Both techniques led to a modest improvement in solubility. Soluplus^®^ enhanced fenbendazole dissolution by acting as a stabilizing carrier, while FT-IR and DSC analysis showed no significant chemical interaction between the drug and polymer. XRPD tests revealed that fenbendazole largely retained its crystalline structure, which contributed to reducing its solubility after drying. However, the presence of Soluplus^®^ significantly reduced the intensity of X-ray diffraction patterns, significantly suggesting decreased crystallinity.

The dissolution studies further demonstrated that microfluidization and ultrasonication enhanced fenbendazole solubility. The enhancement was considerable over multiple cycles, particularly with the inclusion of Soluplus^®^. However, drying caused a significant loss of solubility, which is most probably due to reaggregation, confirmed by DLS analysis results, which showed increased particle sizes in post-drying samples regardless of the applied size reduction techniques, drying methods, or even Soluplus^®^ presence.

## Figures and Tables

**Table 1:** Sample ingredients and preparation methods *.

Sample Name	Fenbendazole Content (mg)	Soluplus^®^ Content (mg)
Fen	Pure	-
Sol	-	Pure
FenMic	200	0
FenMic5FD	200	0
FenMic5SC	200	0
FenSon	200	0
FenSon3FD	200	0
FenSon3SC	200	0
SolFenMic	200	800
SolFenMic5FD	200	800
SolFenMic5SC	200	800
SolFenSon	200	800
SolFenSon3FD	200	800
SolFenSon3SC	200	800

* Sample naming convention: Fen, Sol, Son, Mic, FD, and SC stand for fenbendazole, Soluplus^®^, ultrasonication, microfluidizer, freeze-dryer, and sample concentrator, respectively. Mic5 means 5 cycles of microfluidization, and Son3 indicates 60 min of ultrasonication. Fen and Sol stand for fenbendazole and Soluplus^®^ as the specific neat samples. These names are used in tables and figures to enhance readability.

**Table 2:** The thermal behavior data of fenbendazole and its processed samples extracted from Figure 3 using Pyris Manager software and Origin Pro 2022b, including T_m1_, T_m2_, ΔH_f_, and X_c_, which is calculated using Equation (2).

Sample Name	First T_m_ T_m1_ (°C)	Second T_m_ T_m2_ (°C)	Enthalpy of Fusion (ΔH_f_) J/g	Degree of Crystallinity (X_c_)
Fen	235	-	222.4 *	100.00
FenSon3FD	235	238	220.6	99.36
FenSon3SC	230	236	193.0	86.93
FenMic5FD	230	236	183.5	82.65
FenMic5SC	235	-	159.1	77.61

* Fenbendazole enthalpy of fusion is considered as the enthalpy of fusion for fully crystalline reference (ΔHf˚) in Equation (2).

**Table 3:** Average water solubility and UV absorbance observed at 288 nm of fenbendazole and fenbendazole–Soluplus^®^ samples during the microfluidization cycles.

Microfluidization Cycles	Fen Solubility μg/mL	UV Abs. at 288 nm (Fen)	Fen-Sol Solubility μg/mL	UV Abs. at 288 nm (Fen-Sol)
0	0.000	0.000	0.000	0.000
1	0.395	0.014	0.763	0.027
2	0.734	0.026	2.685	0.095
3	0.961	0.034	9.808	0.347
4	1.158	0.041	12.351	0.437
5	1.187	0.042	12.379	0.438

**Table 4:** Average water solubility and UV absorbance observed at 288 nm of fenbendazole and fenbendazole–Soluplus^®^ samples during the ultrasonication process.

Sonication Time min	Fen Solubility μg/mL	UV Abs. at 288 nm (Fen)	Fen–Sol Solubility μg/mL	UV Abs. at 288 nm (Fen-Sol)
0	0.000	0.000	0.000	0.000
15	0.508	0.018	2.572	0.091
30	0.650	0.023	11.447	0.405
60	1.272	0.045	18.371	0.650
90	1.356	0.048	18.400	0.651

**Table 5:** UV absorbance values of the post-drying samples were measured from three replicate measurements during a 24 h (1440 min) dissolution test in water at 37 ± 1 °C. When the three replicate readings were identical, SD = 0; such samples are indicated by a line beneath the corresponding data. For other samples showing small variation among replicates, the SD was 5.8 × 10^−4^, identical across all sets except those with SD = 0. To maintain clarity and readability, only the mean values are reported.

	Time (min)	0	15	30	60	120	180	240	360	1440
Samples										
Fen		0.0000	0.0053	0.0093	0.0106	0.0106	0.0110	0.0110	0.0106	0.0110
FenMic5FD		0.0000	0.0076	0.0156	0.0213	0.0227	0.0230	0.0230	0.0233	0.0253
FenMic5SC		0.0000	0.0077	0.0143	0.0220	0.0237	0.0240	0.0240	0.0243	0.0256
FenSon3FD		0.0000	0.0087	0.0173	0.0263	0.0266	0.0270	0.0273	0.0270	0.0270
FenSon3SC		0.0000	0.0070	0.0143	0.0230	0.0253	0.0260	0.0266	0.0263	0.0266
SolFenMic5FD		0.0000	0.0090	0.0176	0.0276	0.0286	0.0286	0.0283	0.0283	0.0286
SolFenMic5SC		0.0000	0.0093	0.0203	0.0303	0.0306	0.0306	0.0306	0.0320	0.0320
SolFenSon3FD		0.0000	0.0093	0.0180	0.0280	0.0280	0.0286	0.0290	0.0283	0.0283
SolFenSon3SC		0.0000	0.0100	0.0190	0.0276	0.0313	0.0323	0.0316	0.0320	0.0320

**Table 6:** Solubility values of the post-drying samples measured from three replicate measurements during a 24 h (1440 min) dissolution test in water at 37 ± 1 °C. When the three replicate readings were identical, SD = 0; such samples are indicated by a line beneath the corresponding data. For other samples showing small variation among replicates, the SD was 1.6 × 10^−2^, identical across all sets except those with SD = 0. To maintain clarity and readability, only the mean values are reported.

	Time (min)	0	15	30	60	120	180	240	360	1440
Samples										
Fen		0.0000	0.150	0.263	0.299	0.299	0.311	0.311	0.299	0.311
FenMic5FD		0.0000	0.215	0.441	0.602	0.641	0.650	0.650	0.658	0.715
FenMic5SC		0.0000	0.218	0.405	0.622	0.670	0.678	0.678	0.687	0.723
FenSon3FD		0.0000	0.246	0.489	0.743	0.752	0.763	0.772	0.763	0.763
FenSon3SC		0.0000	0.198	0.405	0.650	0.716	0.734	0.752	0.743	0.752
SolFenMic5FD		0.0000	0.254	0.497	0.782	0.808	0.808	0.799	0.799	0.808
SolFenMic5SC		0.0000	0.263	0.574	0.856	0.864	0.864	0.864	0.904	0.904
SolFenSon3FD		0.0000	0.263	0.509	0.791	0.791	0.808	0.819	0.799	0.799
SolFenSon3SC		0.0000	0.283	0.537	0.780	0.885	0.913	0.893	0.904	0.904

**Table 7:** EDX quantitative elemental analysis.

Samples	Carbon %	Oxygen %	Sulfur %	Others %	Total %
FenSon3SC	88.83	9.17	1.42	0.58	100.00
FenSon3FD	82.04	14.27	2.80	0.89	100.00
FenMic5SC	71.61	24.84	2.45	1.10	100.00
FenMic5FD	73.81	23.40	2.64	0.15	100.00
SolFenSon3SC	73.23	25.41	0.63	0.73	100.00
SolFenSon3FD	80.69	17.05	0.42	1.84	100.00
SolFenMic5SC	69.65	28.03	0.58	1.74	100.00
SolFenMic5FD	80.38	18.89	0.24	0.49	100.00

**Table 8:** Degree of crystallinity of the samples calculated based on the XRPD data.

Sample Name	Degree of Crystallinity (X_c_)
Fen	100.00
FenSon3FD	92.57
FenSon3SC	90.65
FenMic5FD	95.11
FenMic5SC	93.49
SolFenSon3FD	3.76
SolFenSon3SC	3.52
SolFenMic5FD	5.68
SolFenMic5SC	4.71

**Table 9:** Particle size distribution and average particle size of samples after size reduction techniques.

Sample	Ave. Particle Size (d.nm)	PDI *
FenMic5	2215.64	0.188
SolFenMic5	318.20	10.905 *
FenSon3	1820.44	0.632
SolFenSon3	308.54	5.054 *
FenMic5FD	3766.83	0.075
FenMic5SC	3910.75	0.121
FenSon3FD	3585.76	0.145
FenSon3SC	3864.77	0.143
SolFenMic5FD	2126.68	1.480 *
SolFenMic5SC	2569.79	0.641
SolFenSon3FD	1531.52	1.076 *
SolFenSon3SC	958.33	0.404

* In bi-modal dispersity systems, PDI is not usual (PDI > 1); this observation may also be attributed to particle aggregation.

## Data Availability

The data presented in this study are available upon request from the corresponding author.
